# Looking Back to Look Forward: What to Expect in a Redo Surgery for a Bioprosthesis Replacement

**DOI:** 10.3390/jcm11237104

**Published:** 2022-11-30

**Authors:** Ilaria Giambuzzi, Giorgia Bonalumi, Giulia Ballan, Pietro Messi, Alice Bonomi, Analia Maggiore, Giampiero Esposito, Michele Di Mauro, Francesco Alamanni, Marco Zanobini

**Affiliations:** 1 Department of Cardiac Surgery, Centro Cardiologico Monzino IRCCS, 20137 Milan, Italy; 2 I.R.C.C.S. Ospedale Galeazzi—Sant’Ambrogio, 20157 Milan, Italy; 3 Department of Biostatistics, Centro Cardiologico Monzino IRCCS, 20137, Milan, Italy; 4 Cardio-Thoracic Surgery Department, Heart & Vascular Centre, Maastricht University Medical Centre, 6229 HX Maastricht, The Netherlands; 5 Dipartimento Scienze Cliniche e di Comunità (DISCCO Department), University of Milan, 20122 Milan, Italy

**Keywords:** bioprosthesis replacement, redo surgery, structural valve degeneration, endocarditis

## Abstract

Redo surgeries are becoming more common because of an increased rate of bioprosthesis implantation. We performed a retrospective study on patients who underwent redo replacement of an aortic and/or mitral bioprosthesis between 2005 and 2018 to evaluate intra-hospital mortality and morbidity. Univariate analysis was performed on the propensity score variables to determine predictors of mortality. A total of 180 patients were enrolled in the study: Group A (replacement of aortic bioprosthesis) with 136 patients (75.56%) and group B (replacement of mitral bioprosthesis ± aortic bioprosthesis) with 44 patients (24.44%). NYHA class ≥ 3 and female sex were significantly more common in group B. Cardiopulmonary-bypass time and aortic cross-clamping time in group A and group B were, respectively, 154.95 ± 74.35 and 190.25 ± 77.44 (*p* = 0.0005) and 115.99 ± 53.54 and 144.91 ± 52.53 (*p* = 0.0004). Overall mortality was 8.89%. After propensity score adjustment, Group B was confirmed to have an increased risk of death (OR 3.32 CI 95% 1.02–10.88 *p* < 0.0001), gastrointestinal complications (OR 7.784 CI 95% 1.005–60.282 *p* < 0.0002) and pulmonary complications (OR 2.381 CI 95% 1.038–5.46 *p* < 0.0001). At the univariate analysis, endocarditis, cardiopulmonary-bypass and aortic cross clamping time, NYHA class ≥ 3 and urgency setting were significantly associated to death. Intra-hospital outcomes were acceptable regarding mortality and complications. Patients who need redo surgery on mitral bioprosthesis have an increased risk of post-operative pulmonary and gastrointestinal complications and mortality. Therefore the choice of mitral bioprosthesis at time of first surgery should be carefully evaluated.

## 1. Introduction

In cardiac valve surgery, the most commonly used prostheses to replace patients’ diseased valves are biological ones, as opposed to mechanical ones [1,2]. Indeed, bioprostheses have several advantages: first of all, life-time anticoagulant therapy is usually deemed not necessary, even if patients might have the indications to take the anticoagulant drugs for a short period [3]. Secondly, bioprostheses are not noisy, which means that they cause less discomfort to patients. Nevertheless, there are also disadvantages linked to the use of biological valves: firstly, a smaller effective valve orifice and, secondly, the structural degeneration of the prosthesis [4]. This latter mentioned disadvantage is unavoidable at the present time and determines the need to undergo a reoperation [5,6].

The main reason for the rise in valvular reinterventions or redo surgeries is that a growing number of biological prostheses are being implanted in young patients [7], partly due to the development of percutaneous surgeries in recent years, which may allow a future valve-in-valve procedure [8]. Indeed, biological prostheses can also be recommended for patients younger than 50 years old [9,10].

Percutaneous techniques for treating failing valvular bioprostheses are developing more and more but remain an alternative to surgery in the medium-high surgical risk group only for the aortic valve, with the TAVI technique, and for the high surgical risks associated with the more complex mitral valve. A further unknown of transcatheter valve implantation techniques is the durability of the valve bioprostheses [11] and the consequent risk in explanting a TAVR [12].

Nevertheless, redo surgery has higher mortality and morbidity when compared to first surgery [13,14].

The aim of this study is to analyze the immediate post-operative outcomes (survival and main complications) of patients who undergo redo cardiac surgery on a previously implanted bioprothesis through the assessment of a group of patients subjected to the above-mentioned surgical operation.

## 2. Materials and Methods

We performed a retrospective monocentric study on patients who underwent replacement of a bioprosthesis in the aortic and/or mitral position between 2005 and 2018. The study was approved by the local ethical committee (n. R1480/21-CCM 1554) with the need for consent waived given the retrospective nature of the study. Data are available upon request.

Inclusion criteria included previous surgery with implantation of biological prosthesis in aortic and/or mitral positions (in the case of double replacement, both valves were, in all cases, replaced with bioprostheses at the time of first surgery) and indication to undergo redo surgery because of malfunctioning of the valve. Exclusion criteria included only being under age.

Intraoperative data were obtained retrospectively and stored in a database.

The biological prosthesis dysfunction definition has been reviewed over the years. Aetiology for redo surgery was either endocarditis, paravalvular leak or structural valve deterioration (SVD). Our patients who underwent redo surgery because of SVD were in stage 3 of the definition proposed by Dvir Danny et al. [4].

Pulmonary complications were defined as pleural effusion and/or pneumothorax needing tube placement, pneumonia, prolonged mechanical ventilation (>48 h) and acute pulmonary insufficiency (P/F < 100). Gastrointestinal complications were defined as intestinal ischemia or perforation.

### 2.1. Diagnostic Work-Up and Surgery

In the case of elective surgery, all patients underwent echocardiographic studies to evaluate and define the aetiology of the bioprosthesis disease. In the case of endocarditis, an antibiotic therapy was also initiated. Moreover, a CT scan was performed to study adherences and the sternal relationship with the heart.

In contrast, in urgent cases, once the correct diagnosis was obtained, the CT scan might have not been performed, depending on the clinical status of the patient.

The surgery was carried out through re-sternotomy (only one patient underwent thoracotomy for replacement of mitral bioprosthesis) and cardiopulmonary bypass (CPB) was instituted either centrally or peripherally, depending on mediastinal adherences. After aortic cross clamp, the left atrium and/or aorta were opened to examine the bioprosthesis and confirm the indication (SVD, endocarditis or paravalvular leak). Subsequently, the bioprosthesis was removed and a new prosthesis was implanted. In the case of replacement of mitral and aortic bioprostheses, the mitral bioprosthesis was implanted before the aortic one. The choice of the new type of prosthesis (biological or mechanical) was discussed pre-operatively with the patient and decided upon depending on the age, comorbidities and risk of another surgery.

### 2.2. Statistical Analysis

Continuous variables were expressed as mean ± standard deviation, for normally distributed variables, as medians and quartiles (25–75%) for continuous variables not normally distributed and as numbers (percentages) for categorical variables. To identify differences between the two groups in terms of mean, median, or percentage, *t*-test, Wilcoxon’s test, Fisher’s exact test, and χ^2^ were used. The multivariate logistic model was implemented to assess whether the group was a predictor of the individual endpoints (exitus, gastrointestinal complications and pulmonary complications), after adjustment for propensity score. The propensity score was estimated running a logistic model including these characteristics: preoperative ECG, NYHA class, etiology, and endocarditis; these were chosen through an epidemiological approach (i.e., those factors that in the clinician’s experience can be confounders). 

Finally, a univariate analysis was performed on the propensity score variables to determine predictors of mortality (as total intra-hospital death). A *p*-value < 0.05 was considered significant.

All analyses were performed using SAS 9.4 software.

## 3. Results

### 3.1. Pre Operative Results

A total of 180 patients underwent redo surgery between 2005 and 2018, among 8500 who underwent cardiac surgery. Patients were divided in two groups: Group A and Group B. Group A included 136 (75.56%) cases who underwent replacement of an aortic bioprosthesis. Group B included 44 (24.44%) patients who underwent replacement of a mitral valve bioprosthesis only (30 patients) or of both mitral and aortic valve bioprostheses (14 patients). Pre-operative characteristics are reported in Table 1.

The overall mean age was 61 ± 14.97 years old. More specifically group A was 60.79 ± 15.74 years old, while that of group B was 63.41 ± 12.08. 

Among the 180 patients, 111 were male and 69 female. Notably, the proportion of female sex was significantly higher in group B (63.64%) than in group A (30.15%) (*p* < 0.0001). Moreover, 68 patients (37.78%) had an NYHA class ≥ 3, 45 (33.09%) in group A and 23 (52.27%) in group B (*p* = 0.025) (Table 1). The mean telediastolic diameter was in range of normality in both groups. Aetiology for redo surgery is described in Table 1.

Out of 180 patients, 41 (22.78%) underwent surgery because of endocarditis, 125 (69.44%) had a bioprosthesis degeneration and 11 (6.11%) had a paravalvular leak. We did not observe any statistical differences between the two groups.

### 3.2. Intra Operative Results

Intraoperative features taken into account for this study are listed in Table 2.

The number of surgeries performed in an emergency setting were significantly higher in group A (19 patients, 13.96%), than in group B (1 patient, 2.27%) (*p* = 0.03).

Only in 11 patients (6.11%) was cardiopulmonary bypass instituted through femoral vessels. In all other cases, a central cannulation was preferred.

Clamping time was also statistically different between the two groups: 115.99 ± 53.54 min in group A versus 144.91 ± 52.53 in group B (*p* = 0.0004). Lastly, cardiopulmonary bypass (CPB) time was observed to be higher in group B (190.25 ± 77.44) than in group A (154.95 ± 74.35) (*p* = 0.0005).

Of the whole considered population, 30.56% underwent concomitant procedures (including tricuspid valve repair and/or aorto-coronary bypass) but no difference between the two groups was noticed.

### 3.3. Post-Operative Results

Overall, 16 patients (8.89%) out of 180 died after surgery, 8 in group B (18.18%) and 8 in group A (5.88%). Hence, the mortality in group B was statistically higher than in group A (*p* = 0.0001). Among causes of death, seven patients (43.75%) died because of multiorgan failure, one patient (6.25%) because of intestinal ischemia, one patient (6.25%) because of intractable haemorrhage in the operating room and seven patients (43.75%) because of intractable cardiac failure. Moreover, among the 16 deceased patients, 12 (75%) underwent surgery because of endocarditis and 6 (37.56%) were operated on in an urgency setting. Anyway, even without including in the analysis patients who had endocarditis (37, 20.5%), mortality was similar. Indeed, on a total of 143 patients, there were 9 deaths (6.29%), in group A 106 with 5 deaths (4.71%) and in group B 37 patients with 4 deaths (10.81%). 

Post-operative complications are listed in Table 3.

Pulmonary complications affected 45 patients (25%) in total; group B reported a higher percentage of these complications (38.64%) than group A (20.59%) (*p* = 0.0001). GI complications were also higher in group B: 6.82% vs. 1.47% (*p* = 0.0002). After the propensity score adjustment, it was confirmed that patients in group B had a significantly higher risk of mortality, gastrointestinal and pulmonary complications (Table 4).

A univariate analysis was then performed to evaluate potential risk factors for mortality in our whole population. All analyzed variables are listed in Table 5.

## 4. Discussion

In the last few years of valvular surgery, bioprostheses are being implanted more commonly. Therefore, surgeons are facing many redo surgeries to replace a failing biological prosthesis. In order to evaluate the impact of REDO surgery for replacement of biological prosthesis on intra-hospital outcomes, we performed a retrospective study on patients who underwent replacement of aortic and/or mitral biological prosthesis. The aetiologies taken into account were either SVD, endocarditis or paravalvular leak. The overall mortality was 8.89%. Moreover, the results showed that replacement of mitral bioprosthesis was an independent risk factor for death, gastrointestinal and pulmonary complications. 

Our mortality was in line with already published data, being between 7.3% and 10.9% [15,16,17,18,19]. Among predictors of mortality, our univariate analysis found that CPB time, aortic cross-clamping time, NYHA ≥ 3, urgency setting and endocarditis were significant predictive factors. Sex was not a significant predictor, which is in line with literature, which shows conflicting results. Indeed, Vogt et al. [20] and Pansini et al. [18] found a higher mortality in females, while Akins et al. [17] reported an increased mortality in males. Longer CPB time and aortic cross-clamping times are known risk factors for worse surgical outcomes, as are urgency and endocarditis. Indeed, 43.7% of exitus was present in patients who underwent surgery for endocarditis, which is also in line with previously published studies [17,19]. Moreover, a higher NYHA class is known to be a risk factor for mortality [7,19,20].

Of interest, replacement of mitral bioprosthesis was a risk factor for death, gastrointestinal and pulmonary complications. A similar result was reported by Jones et al. and Lytle et al. [13,14,15,16,17,18,19], whose studies demonstrated that patients undergoing mitral valve replacement have a higher risk of mortality than patients undergoing aortic valve replacement, mainly due to post-operative acute myocardial infarction, rupture of the left ventricle and arrhythmias. Nevertheless, the causes of death in our patients in group B were multi-organ failure and cardiogenic shock. The increased mortality in patients who underwent a replacement of a mitral bioprosthesis might have both surgical and clinical reasons. First of all, surgical access to the mitral valve requires a deeper lysis of adherences and an increased manipulation of the heart. Furthermore, patients are usually more frail. Indeed, patients in group B were more prone to have an NYHA ≥ 3, indicating a worse underlying clinical state [21].

Replacement of a mitral valve bioprosthesis resulted in an increased risk of gastrointestinal complications. In the literature [17,22,23,24], causes are mainly related to low cardiac output syndrome, post-operative arrhythmias (no difference in our pool of patients), use of noradrenaline and intra-aortic balloon pump (which had a higher incidence in group B in our study) and CPB time (significantly higher in group B). Moreover, Balsam et al. [21] showed a correlation between NYHA ≥ 3 and gastrointestinal complications, in line with our results. Pulmonary complications might also be related to the worse clinical picture of the patients in group B because of the underlying pathology. Mitral pathology already causes an altered lung function, and it could be exacerbated in a redo surgery.

Our study shows that a redo surgery to change a biological prosthesis is not risk-free. Despite advent of valve-in-valve procedures, their use might not be indicated in all cases, such as endocarditis, risk of patient–prosthesis mismatch, high risk of embolization and left ventricle tract obstruction. Moreover, long term results are still lacking. Therefore, the role of the heart team during the first surgery becomes pivotal in assess, as much as possible, the possibility of permitting a future valve-in-valve procedure and the risk of a future re-intervention.

The most important limitation of our study is its retrospective nature. Moreover, it covers a long span of time, therefore developments in surgical techniques have been made during it. It lacks a long term follow up, even though this was not the primary objective of the study, it could give a deeper insight to the results of the surgery.

## 5. Conclusions

In our experience, redo surgery for replacement of mitral bioprosthesis carries an increased risk of mortality and serious complications (gastrointestinal and pulmonary). Therefore, the choice of biological prosthesis at the time of first surgery must be carefully evaluated, and the anatomical criteria for a future percutaneous mitral valve- in-valve procedure might be assessed at the time of the first surgery, in order to assess the possibility of performing a minimally invasive treatment for a future prosthesis dysfunction. Nevertheless, the patients should be aware that, in case of a bioprosthesis dysfunction needing a traditional open surgery (which might anyway be the only option, especially in case of endocarditis), mitral bioprosthesis replacement carries an higher risk in terms of mortality, gastrointestinal and pulmonary complications when compared to other standard redo valvular surgery.

## Figures and Tables

**Table 1:** Pre operative characteristics.

	N°	Group A	Group B	*p*-Value
Total number of patients	180 (100)	136 (75.56)	44 (24.44)	
Age (years)	61 ± 14.97	60.79 ± 15.74	63.41 ± 12.08	0.316
Female sex	69 (38.33)	41 (30.15)	28 (63.64)	<0.0001
Euroscore II	10.04 ± 11.02	9.13 ± 10.47	10.96 ± 12.61	0.188
Arterial Hypertension (>140/90 mmHg)	101 (56.11)	77 (56.62)	24 (54.55)	0.809
Chronic kidney disease	124 (68.89)	93 (68.38)	31 (70.45)	0.4932
BMI	25.5 ± 3.43	25.5 ± 3.43	25.44 ± 3.5	0.003
Smoke	71 (39.44)	54 (39.71)	17 (38.64)	0.98
Cerebrovascular disease	22 (12.15)	15 (11.02)	7 (15.09)	0.07
History of AMI	13 (7.22)	8 (5.88)	5 (11.36)	0.222
Diabetes	29 (16.11)	21 (15.44)	8 (18,18)	0.59
COPD	19 (10.56)	15 (11.03)	4 (9.09)	0.716
Dyslipidemia	94 (52.22)	70 (51.47)	24 (54.55)	0.723
Peripheral vascular disease	18 (10.00)	13 (9.56)	5 (11.36)	0.7286
NYHA ≥ 3	68 (37.78)	45 (33.09)	23 (52.27)	0.025
EF	57.06 ± 10.46	57.87 ± 9.95	56.25 ± 11.91	0.4649
TDD (mm)	51.91 ± 10.41	52.5 ± 9.39	50.28 ± 8.84	0.203
Endocarditis	37 (20.55)	30 (22.06)	7 (15.91)	0.6786
Bioprosthesis degeneration	131 (72.78)	97 (71.32)	34 (77.27)	
Paravalvular leak	12 (6.67)	9 (6.61)	3 (6.82)	

Values are reported as *n* (%) if categorical variable or mean ± standard if continuous variable. BMI: body mass index; AMI: acute myocardial infarction; chronic kidney disease: kidney damage (structural/functional abnormalities, GFR < 60 mL/min/1.73 m^2^, ≥months); COPD: chronic obstructive pulmonary disease; EF: ejection fraction; TDD: telediastolic diameter.

**Table 2:** Intra operative characteristics.

	N°	Group A	Group B	*p*-Value
Time to redo (days),		2989.57 ± 2097.66	3030.84 ± 1953.00	0.4381
Emergent surgery	20 (11.11)	19 (13.96)	1 (2.27)	0.03
Aortic cross clamping time (min)	123.06 ± 54.73	115.99 ± 53.54	144.91 ± 52.53	0.0004
CPB time (min)	163.58 ± 76.64	154.95 ± 74.35	190.25 ± 77.44	0.0005
IABP, *n* (%)	6 (3.33)	3 (2.21)	3 (6.82)	0.1385
Concomitant procedures	55 (30.56)	41 (30.15)	14 (31.82)	0.836
Biological prosthesis	130 (72.22)	101 (74.26)	29 (65.91)	0.2821
Size of aortic biological prosthesis	23 (21–25)	23 (21–25)	21 (19–23)	0.825
Size of aortic mechanical prosthesis	21 (19–23)	21 (19–23)	19 (19–21)	0.76
Size of mitral biological prosthesis	27 (25–27)	//	27 (25–27)	//
Size of mitral mechanical prosthesis	27 (27–29)	//	27 (27–29)	//

Values are reported as *n* (%) if categorical variable or mean ± standard/median (IQR) if continuous variable. CPB: cardiopulmonary bypass; IABP: intra-aortic balloon pump.

**Table 3:** Post-operative complications.

	N°	Group A	Group B	*p*-Value
Death	16 (8.89)	8 (5.88)	8 (18.18)	0.0001
Neurological complications	14 (7.77)	9 (6.6)	5 (11.36)	0.676
IMA	1 (0.56)	1 (0.74)	0 (0.00)	0.568
ECMO	2 (1.11)	0 (0.00)	2 (4.55)	0,9371
Pulmonary complications	45 (25)	28 (20.59)	17 (38.64)	0.0001
Arrhythmias	48 (26.67)	35 (25.74)	13 (29.54)	0.619
PM implant	17 (9.44)	14 (10.29)	3 (6.82)	0.493
Gastrointestinal complications	5 (2.78)	2 (1.47)	3 (6.82)	0.0002
Acute kidney disease, *n* (%)	85 (47.22)	65 (47.79)	20 (45.45)	0.068
Re-exploration for bleeding	20 (11.11)	17 (12.50)	3 (6.82)	0.2972
LOS ICU (days)	4.62 ± 8.83	4.04 ± 5.33	6.43 ± 15.05	0.0919
LOS (days)	14.23 ± 13.63	13.98 ± 11.61	15.02 ± 18.52	0.7685
Prolonged LOS (>14 days)	51 (28.33)	40 (29.41)	11 (25.00)	0.471

Values are reported as *n* (%) if categorical variable or mean ± standard if continuous variable. Acute kidney disease: KDIGO parameters; AMI: acute myocardial infarction; arrhythmias: atrial fibrillation, atrial flutter, ventricular tachycardia; ECMO: extracorporeal membrane oxygenation; PM: pacemaker; LOS: length of stay; ICU: intensive care unit.

**Table 4:** Propensity score adjustment.

Variable	OR	95% Confidence Interval	*p* Value
Exitus	3.32	1.02–10.88	<0.0001
Gastrointestinal complications	7.784	1.005–60.282	<0.0002
Pulmonary complications	2.381	1.038–5.46	<0.0001

**Table 5:** Univariate regression analysis.

Variable	OR	95% Confidence Interval		*p* Value
Female sex	0.592	0.211	1.659	0.3188
Pre-operative rhythm	0.663	0.308	1.427	0.2931
NYHA ≥ 3	0.363	0.183	0.721	0.0038
CPB time	0.987	0.98	0.993	<0.0001
Aortic cross clamping time	0.987	0.979	0.994	0.0009
Urgency setting	0.156	0.049	0.492	<0.0001
Endocarditis	0.288	0.099	0.834	0.0218

CPB: cardiopulmonary bypass.

## Data Availability

Raw data are available at https://zenodo.org/record/7142169 (link Zenodo) Accessed on 29 November 2022.

## References

[1] Chiang Y.P., Chikwe J., Moskowitz A., Itagaki S., Adams D.H., Egorova N.N. (2014). Survival and Long-term Outcomes Following Bioprosthetic vs Mechanical Aortic Valve Replacement in Patients Aged 50 to 69 Years. JAMA.

[2] Chikwe J., Chiang Y.P., Egorova N.N., Itagaki S., Adams D.H. (2015). Survival and Outcomes Following Bioprosthetic vs Mechanical Mitral Valve Replacement in Patients Aged 50 to 69 Years. JAMA.

[3] Kueri S., Kari F.A., Fuentes R.A., Sievers H.-H., Beyersdorf F., Bothe W. (2019). The Use of Biological Heart Valves: Types of Prosthesis, Durability and Complications. Dtsch. Ärzteblatt Int..

[4] Dvir D., Bourguignon T., Otto C.M., Hahn R.T., Rosenhek R., Webb J.G., Treede H., Sarano M.E., Feldman T., Wijeysundera H. (2018). Standardized Definition of Structural Valve Degeneration for Surgical and Transcatheter Bioprosthetic Aortic Valves. Circulation.

[5] Weber A., Noureddine H., Englberger L., Dick F., Gahl B., Aymard T., Czerny M., Tevaearai H., Stalder M., Carrel T.P. (2012). Ten-year comparison of pericardial tissue valves versus mechanical prostheses for aortic valve replacement in patients younger than 60 years of age. J. Thorac. Cardiovasc. Surg..

[6] McClure R.S., Narayanasamy N., Wiegerinck E., Lipsitz S., Maloney A., Byrne J.G., Aranki S.F., Couper G.S., Cohn L.H. (2010). Late Outcomes for Aortic Valve Replacement with the Carpentier-Edwards Pericardial Bioprosthesis: Up to 17-Year Follow-Up in 1000 Patients. Ann. Thorac. Surg..

[7] Briffa N., Chambers J.B. (2017). Biological Valves in Younger Patients Undergoing Aortic Valve Replacement. Circulation.

[8] Webb J.G., Murdoch D.J., Alu M.C., Cheung A., Crowley A., Dvir D., Herrmann H.C., Kodali S.K., Leipsic J., Miller D.C. (2019). 3-Year Outcomes After Valve-in-Valve Transcatheter Aortic Valve Replacement for Degenerated Bioprostheses: The PARTNER 2 Registry. J. Am. Coll. Cardiol..

[9] Beyersdorf F., Vahanian A., Milojevic M., Praz F., Baldus S., Bauersachs J., Capodanno D., Conradi L., De Bonis M., De Paulis R. (2021). 2021 ESC/EACTS Guidelines for the management of valvular heart disease. Eur. J. Cardio-Thoracic Surg..

[10] Harky A., Suen M.M.Y., Wong C.H.M., Maaliki A.R., Bashir M. (2019). Bioprosthetic Aortic Valve Replacement in <50 Years Old Patients—Where is the Evidence?. Braz. J. Cardiovasc. Surg..

[11] Costa G., Criscione E., Todaro D., Tamburino C., Barbanti M. (2019). Long-term Transcatheter Aortic Valve Durability. Interv. Cardiol. Rev. Res. Resour..

[12] Bapat V.N., Zaid S., Fukuhara S., Saha S., Vitanova K., Kiefer P., Squiers J.J., Voisine P., Pirelli L., von Ballmoos M.W. (2021). Surgical Explantation After TAVR Failure. JACC: Cardiovasc. Interv..

[13] Jones J.M., O’Kane H., Gladstone D.J., Sarsam M.A., Campalani G., MacGowan S.W., Cran G.W. (2001). Repeat heart valve surgery: Risk factors for operative mortality. J. Thorac. Cardiovasc. Surg..

[14] Cohn L.H., Aranki S.F., Rizzo R.J., Adams D.H., Cogswell K.A., Kinchla N.M., Couper G.S., Collins J.J. (1993). Decrease in operative risk of reoperative valve surgery. Ann. Thorac. Surg..

[15] Joshi Y., Achouh P., Menasché P., Fabiani J.N., Berrebi A., Carpentier A., Jouan J. (2018). Multiple reoperations on the aortic valve: Outcomes and implications for future potential valve-in-valve strategy. Eur. J. Cardio-Thorac. Surg..

[16] Potter D.D., Sundt III T.M., Zehr K.J., Dearani J.A., Daly R.C., Mullany C.J., Orszulak T.A. (2005). Operative risk of reoperative aortic valve replacement. J. Thorac. Cardiovasc. Surg..

[17] Akins C.W., Buckley M.J., Daggett W.M., Hilgenberg A.D., Vlahakes G.J., Torchiana D.F., Madsen J.C. (1998). Risk of Reoperative Valve Replacement for Failed Mitral and Aortic Bioprostheses. Ann. Thorac. Surg..

[18] Pansini S., Ottino G., Forsennati P.G., Serpieri G., Zattera G., Casabona R., di Summa M., Villani M., Poletti G.A., Morea M. (1990). Reoperations on heart valve prostheses: An analysis of operative risks and late results. Ann. Thorac. Surg..

[19] Lytle B.W., Cosgrove D.M., Taylor P.C., Gill C.C., Goormastic M., Golding L.R., Stewart R.W., Loop F.D. (1986). Reoperations for Valve Surgery: Perioperative Mortality and Determinants of Risk for 1000 Patients, 1958–1984. Ann. Thorac. Surg..

[20] Vogt P.R., Brunner-LaRocca H.P., Sidler P., Zünd G., Truniger K., Lachat M., Turina M.I. (2000). Reoperative surgery for degenerated aortic bioprostheses: Predictors for emergency surgery and reoperative mortality. Eur. J. Cardio-Thorac. Surg..

[21] Balsam L.B., Grossi E., Greenhouse D., Ursomanno P., DeAnda A., Ribakove G.H., Culliford A.T., Galloway A.C. (2010). Reoperative Valve Surgery in the Elderly: Predictors of Risk and Long-Term Survival. Ann. Thorac. Surg..

[22] Goldstone A.B., Chiu P., Baiocchi M., Lingala B., Patrick W.L., Fischbein M.P., Woo Y.J. (2017). Mechanical or biologic prostheses for aortic-valve and mitral-valve replacement. N. Engl. J. Med..

[23] Gulkarov I., Trocciola S.M., Yokoyama C.C., Girardi L.N., Krieger K.K., Isom O.W., Salemi A. (2014). Gastrointestinal complications after mitral valve surgery. Ann. Thorac. Cardiovasc. Surg..

[24] Ngaage D.L., Cowen M.E., Griffin S., Guvendik L., Cale A.R. (2007). The impact of symptom severity on cardiac reoperative risk: Early referral and reoperation is warranted. Eur. J. Cardio-Thoracic Surg..

